# Dr. J. S. Jewell

**Published:** 1887-05

**Authors:** 


### JEWELL.

DR. JAMES STEWART JEWELL died at his residence in Chicago, on April 18th, aged 49 years and 7 months. At the time of his death he was Emeritus Professor of Nervous and Mental Disease in the Chicago Medical College, having filled a chair in the college for many years prior to the failure of his health. He was a man of exceptional learning, and had a wide reputation as a neurologist. He established and conducted for many years the *Journal of Nervous and Mental Disease*, until ill-health compelled him to relinquish his editorial work and interrupted his professional occupation. Later his health improved some, and he resumed the practice of his profession and started the *Neurological Review*. But a few numbers of this journal had been issued when he had to suspend its publication, because his impaired health would not permit his continuance of the work in connection with it.

His death affords another illustration of what is so often seen in the medical profession—men sacrificing their health and their lives by over-work, against which they admonish their patients, but which advice they disregard themselves.

The death of Dr. Jewell is a loss to our city and the medical profession, as well as to the science of neurology.

